# Nostalgia enhances detection of death threat: neural and behavioral evidence

**DOI:** 10.1038/s41598-021-91322-z

**Published:** 2021-06-16

**Authors:** Ziyan Yang, Constantine Sedikides, Keise Izuma, Tim Wildschut, Emiko S. Kashima, Yu L. L. Luo, Jun Chen, Huajian Cai

**Affiliations:** 1 Institute of Psychology, CAS Key Laboratory of Behavioral Science, 16 Lincui Road, Beijing, 100101 People’s Republic of China; 2 Psychology Department, University of Chinese Academy of Sciences, Beijing, 100049 People’s Republic of China; 3 Center for Research on Self and Identity, School of Psychology, University of Southampton, Southampton, UK; 4 School of Economics and Management, Kochi University of Technology, Kochi, Japan; 5 School of Psychology and Public Health, La Trobe University, Melbourne, Australia

**Keywords:** Human behaviour, Cognitive neuroscience, Emotion

## Abstract

An experiment examined the potency of nostalgia—a sentimental longing for one’s past—to facilitate detection of death-related stimuli, using functional magnetic resonance imaging (fMRI) and behavioral techniques (i.e., judgmental accuracy, reaction times). We hypothesized and found that, at the neural level, nostalgic (relative to control) participants evinced more intense activation in right amygdala in response to death-related (vs. neutral) words. We also hypothesized and found that, at the behavioral level, nostalgic (relative to control) participants manifested greater accuracy in judging whether two death-related (vs. neutral) words belonged in the same category. Exploratory analyses indicated that nostalgic (relative to control) participants did not show faster reaction times to death-related (vs. neutral) words. In all, nostalgia appeared to aid in death threat detection. We consider implications for the relevant literatures.

## Introduction

Nostalgia, defined as “a sentimental longing or wistful affection for the past” (The New Oxford Dictionary of English, 1998, p. 1266), is a self-relevant, bittersweet—albeit predominantly positive—and social emotion^1,2,3^. It arises from tender, warm, fond, and joyful memories of one’s past, mixed with yearning and even sadness for the past’s unattainability. These memories refer to momentous events from one’s life (e.g., anniversaries, birthdays, graduations, vacations), replete with social exchanges between the self and close others (e.g., family members, friends, partners, classmates)^4,5,6^. Nostalgia is prevalent, occurring across ages^7,8,9^, and universal, occurring across cultures^10,11,12^.

We adopt a functional approach in this article. Nostalgia is also an existential emotion^13^. As such, we are interested in the existential function of the emotion, and specifically in the link between nostalgia and existential threat. Detection of threatening signals^14,15^, especially death-related ones^16,17^, is vital for organismic survival. What is the role of nostalgia in the detection of death-related stimuli (i.e., words)? We addressed this question in an experiment involving both a neural index (i.e., functional magnetic resonance imaging or fMRI) and two behavioral indices (i.e., judgmental accuracy, reaction times).

### Does nostalgia aid in the detection of death threat?

Nostalgia invigorates approach motivation. An example is research that manipulates nostalgia and then records initiation of the Behavioral Activation System^18^ (e.g., “I go out of my way to get things I want”), which is an operationalization of approach motivation. Results showed that nostalgia engages the Behavioral Activation System, and also disrupts the Behavioral Inhibition System^19^. The approach-oriented character of nostalgia has been documented in additional empirical streams. For example, nostalgia increases the sense of alertness and energy, instils a sense of youthfulness, fosters openness and creativity, boosts inspiration and optimism, and encourages risk-taking as well as the pursuit of one’s important goals^20,21,22^. We reasoned that, if nostalgia spurs an approach orientation, including alertness and openness, the emotion ought to facilitate the detection of threat.

### The present research

We manipulated nostalgia (compared to control) via briefly presented pictures, and then exposed participants to death-related words and neutral words. In particular, participants judged whether two (death-related vs. neutral) words belonged in the same category^23^. Next, we assessed (1) the neural processes involved in the detection of death-related (vs. neutral) words, using fMRI, (2) the extent to which participants detected accurately the death-related (vs. neutral) words, and (3) participants’ reaction times to the death-related (vs. neutral) words.

#### Neural level

We were concerned with nostalgia’s influence on neural responding to death-related (vs. neutral) words. We focused on activity in amygdala, a region that plays a key role in threat detection^24,25^. The amygdala manifests increased activation during attention to, and detection of, threatening stimuli^26,27^, and patients with bilateral amygdala damage do not show fear and adaptive behavior in response to a threat^28^. Previous research has indicated that mortality salience, an experimentally induced state of heightened concerns for death, instigates more vigorous activity in right amygdala compared to a control condition^29^. Due to nostalgia’s approach orientation or alertness potential, we hypothesized that, in the nostalgia (relative to control) condition, death-related (vs. neutral) words would evoke particularly strong right amygdala activation.

#### Behavioral level

We were also concerned with nostalgia’s impact on behavioral responding to death-related (vs. neutral) words. Our main interest was judgmental accuracy. We hypothesized that nostalgia, by giving rise to an approach orientation or alertness, would increase the detection accuracy of death-related (vs. neutral) words. Specifically, compared to their control counterparts, nostalgic participants would be more accurate when judging whether two death-related (vs. neutral) words belonged to the same category.

As a way of exploration, we also recorded reaction times for participants’ accuracy judgments. We were unable to offer hypotheses about nostalgia’s effects on reaction times, due to doubts about the diagnostic utility of this measure in the pertinent context. Prior research has reported dissociations between neural responses and reaction times^30,31,32^, and between accuracy and reactions times toward threatening information^33,34^.

## Results

### Manipulation check

As intended, participants in the nostalgia condition (*M* = 2.83, *SD* = 0.84) reported feeling more nostalgic than those in the control condition (*M* = 2.13, *SD* = 0.68), *t*(38) = 2.90, *p* = 0.006, 95% confidence interval (CI) [0.21, 1.19], *d* = 0.92. The manipulation was effective.

### fMRI

#### ROI results

**Figure 1:**
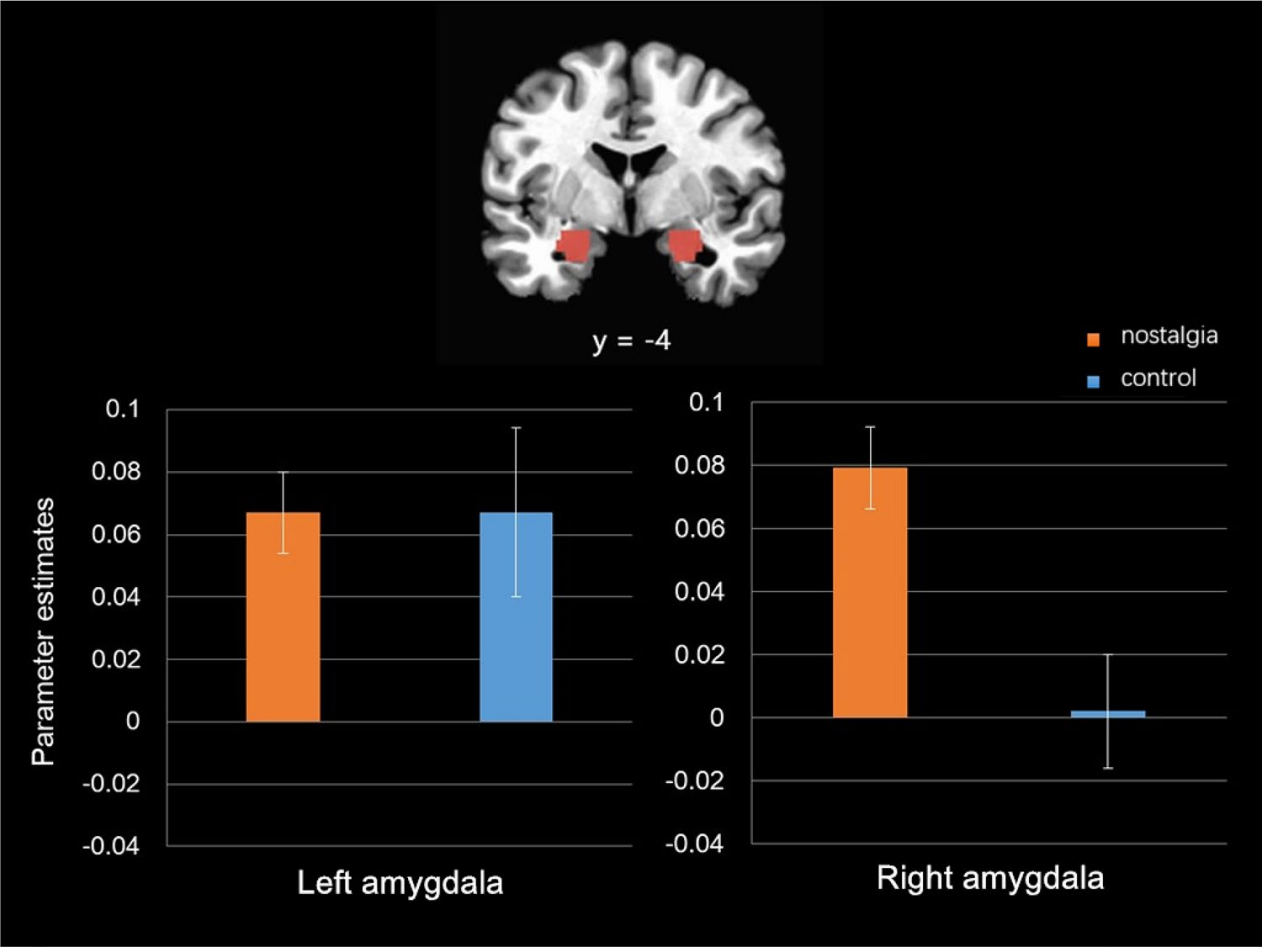
Results of the ROI analyses on left and right amygdala (defined by anatomical regions). Νostalgic (relative to control) participants showed greater right amygdala (but not left amygdala) activation in response to death-related versus neutral words. Error bars show standard errors.

Replicating a previous study^29^, the ROI analysis—including all participants—revealed stronger activity in response to death-related stimuli (vs. neutral stimuli) in right amygdala, *t*(39) = 3.18, *p* = 0.003, 95% CI [0.01, 0.07], *d* = 0.51, but also in left amygdala, *t*(39) = 4.51, *p* < 0.001, 95% CI [0.04, 0.10], *d* = 0.71. More importantly, consistent with our hypothesis, compared with those in the control condition, participants in the nostalgia condition showed enhanced activity in response to death-related (vs. neutral) stimuli in right amygdala, *t*(38) = 3.41, *p* = 0.002, 95% CI [0.03, ,12], *d* = 1.10, but not in left amygdala, *t*(38) = -0.02, *p* = 0.98, 95% CI [-0.06, 0.06] (Fig. 1). Nostalgia intensified the right amygdala response to death-related (vs. neutral) stimuli.

#### Whole brain results

We summarize, in Table 1, the results of the whole brain analyses (with FDR correction) for all participants during the Word Relationship Task. For the contrast of death-related words versus neutral words, we observed significant activation in left and right amygdala, and left inferior frontal gyrus. We also examined the contrast of death-related versus neutral words in the nostalgia condition relative to the control condition (uncorrected for multiple comparisons). In the nostalgia (compared to control) condition, we observed significantly greater activation in right amygdala, left putamen, right anterior cingulate, right middle frontal gyrus, right posterior cingulate, right middle temporal gyrus, and right thalamus in response to death-related (compared to neutral) words (Table 2). The findings converged in showing that nostalgia amplified activity in right amygdala, induced by death threat.

**Table 1:** Brain regions exhibiting significant activation during the word relationship task (death-related words vs. neutral words).

Region	MNI coordinates			Cluster size	*Z*
	x	y	z		
**Death > neutral**					
Left amygdala	− 30	− 28	− 21	349	3.95
Right amygdala	28	− 32	− 24	78	3.46
Left inferior frontal gyrus	− 46	28	− 28	32	3.52

*p* < 0.05; FDR-corrected.

**Table 2:** Brain regions exhibiting significant activation during the word relationship task (death-related words vs. neutral words) in the nostalgia and control conditions.

Region	MNI coordinates			Cluster size	*Z*
	x	y	z		
**Nostalgia > control**					
Right amygdala	24	1	− 16	101	3.37
Left putamen	− 27	− 2	− 9	25	2.98
Right anterior cingulate	15	30	9	16	3.10
Right middle frontal gyrus	28	35	37	16	3.09
Right posterior cingulate	28	− 57	22	13	3.02
Right middle temporal gyrus	43	− 72	34	13	2.91
Right thalamus	8	− 9	10	11	3.01

Death-related words > Neutral words; *p* < 0.005 (uncorrected); min. cluster = 10.

#### Summary

Death-related (vs. neutral) stimuli elicited greater brain activity in right (and left) amygdala among all participants, in line generally with prior findings^29^. More importantly, the activity in right amygdala was stronger in the nostalgia than the control condition. Given that amygdala is typically involved in the processing of threat-related stimuli^24,25^, these findings suggest that nostalgia facilitates detection of death threat.

### Accuracy

During the Word Relationship Task, participants viewed death-related, neutral, and mixed (control) word pairs. They were instructed to judge whether the two words in each pair belonged to the same category. In accordance with prior practice^23^, we analyzed accuracy, that is, the rate of correct responses (i.e., *Same Category*) to death-related and neutral word pairs (excluding control pairs). In particular, we conducted a 2 (nostalgia: nostalgia, control) × 2 words (death-related, neutral) Analysis of Variance (ANOVA) on accuracy.

The interaction was trending, *F*(1, 38) = 4.06, *p* = 0.051,$\eta_{p}^{2}$ = 0.097. We proceeded with tests of simple effects (using the overall error term), given our hypothesis. Nostalgic participants were significantly more accurate in responding to death-related words (*M* = 96.07%, *SD* = 4.62%) versus neutral words (*M* = 91.43%, *SD* = 10.77%), *F*(1, 38) = 5.04, *p* = 0.031, $\eta_{p}^{2}$ = 0.117, whereas control participants were equivalently accurate in responding to death-related words (*M* = 95.36%, *SD* = 5.06%) and neutral words (*M* = 96.61%, *SD* = 5.73%), *F*(1, 38) = 0.37, *p* = 0.55, $\eta_{p}^{2}$ = 0.010.

The words main effect was not significant, *F*(1, 38) = 1.35, *p* = 0.25, $\eta_{p}^{2}$ = 0.034. Some studies have reported a null main effect, like ours^23,35^, whereas another study reported lower accuracy for death-related (vs. neutral) stimuli^31^. Lastly, the nostalgia main effect was not significant either, *F*(1, 38) = 1.81, *p* = 0.187, $\eta_{p}^{2}$ = 0.045.

### Reaction times

We conducted a 2 (nostalgia: nostalgia, control) × 2 words (death-related, neutral) ANOVA on reaction times of correct responses in the Word Relationship Task. The crucial interaction was not significant, *F*(1, 38) = 1.59, *p* = 0.21, $\eta_{p}^{2}$ = 0.040, and nor was the words main effect, *F*(1, 38) = 0.82, *p* = 0.37, $\eta_{p}^{2}$ = 0.021. The relevant literature is conflicting, with some studies reporting null effects like ours^23,35^ and another study reporting longer reaction times for death-related (vs. neutral) stimuli^36^. Finally, the nostalgia main effect was significant: Nostalgic participants (*M* = 1.11 s, *SD* = 0.38 s) manifested slower reaction times than control participants (*M* = 0.89 s, *SD* = 0.16 s), *F*(1, 38) = 5.71, *p* = 0.022,$\eta_{p}^{2}$ = 0.131.

## Discussion

We collected neural and behavioral evidence for the role of nostalgia in detecting death-related stimuli. The results were largely consistent with our hypotheses. Nostalgic (relative to control) participants exhibited stronger activation in right amygdala in response to death-related (vs. neutral) words. Also, nostalgic (relative to control) participants manifested greater accuracy for death-related (vs. neutral) word categorizations, suggesting that nostalgia enhances the detection accuracy of death-related stimuli. Nostalgic participants did not evince faster reaction times; rather, they manifested slower reaction times when categorizing death-related and neutral words.

This research is the first to show that nostalgia likely modulates the processing of information about death. The fMRI results indicated that nostalgia intensified activity in brain regions mainly involved in threat detection, as a function of mortality salience. Those brain regions concern amygdala. Indeed, increased amygdala activation marks the automatic detection of threatening stimuli^14,24^, whereas damage to amygdala impairs the ability to detect potential threat^27^. The facilitating role of nostalgia in the detection of death threat has evolutionary implications. Threat processing is critical for organismic survival and reproduction^15,37^. Potential threats are usually automatically detected, activating subcortical structures such as amygdala, and initiating protective fear responses^14^. Nostalgia strengthens threat processing by amplifying amygdala activation to death-related stimuli.

More broadly, amygdala plays a role in integrating relevant sensory information with emotionally salient memories^38,39^. Nostalgia strengthens amygdala activity, suggesting that nostalgia leads to more in-depth processing of death-related stimuli, enabling emotionally significant experiences to be well processed and remembered. Such processes may be useful in subsequent coping with death threat^13,40^. Along these lines nostalgia facilitated correct responses to death-related words as well.

Our study constitutes the first attempt to examine whether nostalgia facilitated detection of death-related stimuli. As such, our results would need to be replicated with bigger samples, although our study had adequate power to detect the effects of interest. Also, the results would need to be replicated with a stronger manipulation of nostalgia. Although our manipulation was successful, such that participants in the experimental condition reported higher levels of experienced nostalgia than those in the control condition, the mean nostalgia level in the experimental condition was relatively low (although it did significantly exceed the scale midpoint).

In conclusion, the results, albeit preliminary, appear congruent with the role of nostalgia as an approach-oriented emotion. Nostalgia may facilitate the detection of death-related threat. We hope the findings spark investigations into the issue.

## Method

We obtained informed consent from all participants according to the Declaration of Helsinki. Our research was approved by the Institutional Review Board of Institute of Psychology, Chinese Academy of Sciences.

### Participants and design

An a priori power analysis (G*Power 3.1^41^), based on an effect size from a prior relevant behavioral experiment^40^ (*d* = 0.79; Study 3), suggested that 42 participants were needed to ensure 80% statistical power. For logistical reasons, we had to conclude our study at 40 participants. We proceeded to conduct a post-hoc power analysis (G*Power 3.1^41^) based on the effect size of our main finding (i.e., *d* = 1.10). The study achieved 96% statistical power.

We tested 40 undergraduate students (22 women, 18 men; age in years: *Range* = 18–26, *M* = 22.24, *SD* = 2.11) from 12 Beijing-based universities (e.g., Beijing Forestry University, Beijing Normal University, University of Chinese Academy of Sciences). We remunerated each student with 120 RMB (≈ £14). We randomly assigned participants to the nostalgia (*n* = 20) or control (*n* = 20) condition. All of them were right-handed (self-reported), had normal vision (with correction), and were free of regular use of any substance that might influence the central nervous system. Also, none had a history of neurological disease.

The day before the formal experiment commenced, we measured trait nostalgia with the Southampton Nostalgia Scale^3^ (alpha = 0.74) and trait self-esteem with the Rosenberg Self-Esteem Scale^42^ (alpha = 0.90), as evidence has indicated that these two traits are relevant to death threat processes^23,40^. We obtained no differences between the nostalgia and the control conditions on these two traits on any measure (*ps* > 0.20). Also, we obtained no gender and age differences between the nostalgia and the control conditions on any measure (*ps* > 0.53).

### Stimuli and procedure

**Figure 2:**
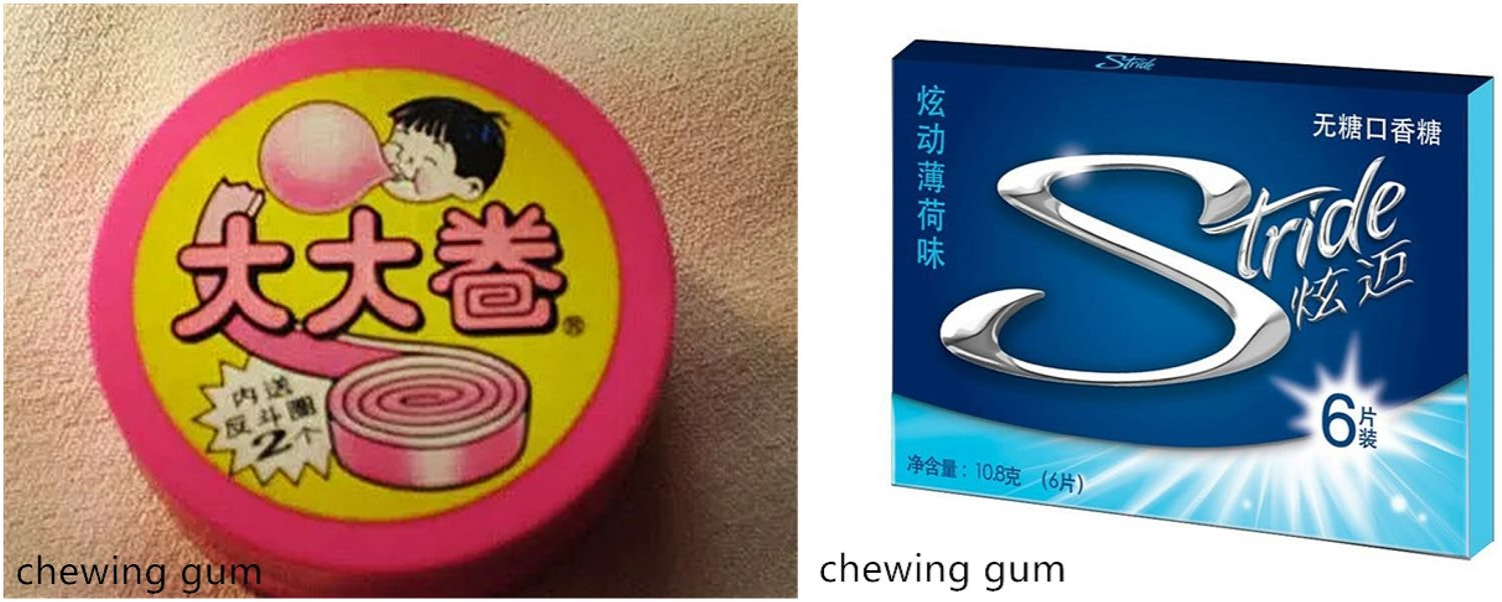
Examples of stimuli (i.e., chewing gum): nostalgia (left) and control (right). The left object depicts the popular chewing gum from childhood; the right object depicts the contemporary popular chewing gum.

During the fMRI scanning, participants completed the nostalgia manipulation and Word Relationship Task. The nostalgia manipulation came first. We relied on a visual stimuli induction method introduced by Oba and colleagues^43^ in their fMRI experiment. We started by generating 26 nostalgic pictures, 26 control pictures, and 26 baseline pictures. The nostalgic pictures depicted scenes or objects from childhood, whereas the control pictures depicted equivalent scenes or objects from modern life. For example, the nostalgic pictures depicted an elementary school classroom, an object (chewing gum), or a cartoon that referred to participants’ childhood. The control pictures, by contrast, depicted a contemporary classroom, chewing gum, or cartoon. The baseline pictures were neutral, non-nostalgic pictures. We present relevant details from pilot testing in Supplementary Material, and an example of a nostalgic and control picture in Fig. 2. The complete set of stimulus materials is available from the authors upon request.

Participants in the nostalgia condition viewed 26 nostalgic pictures, each accompanied (before or after) by one and the same baseline picture. Participants in the control condition viewed 26 control pictures, each accompanied (before or after) by one baseline picture. The nostalgia and control pictures were presented in separate random orders for each participant. Pictures were presented for 8 s each, with an inter-stimulus interval of 8 s. During that interval, a fixation cross appeared on the screen. We instructed participants to focus attentively on the screen. At the end of the session, participants completed a 3-item manipulation check^4,6^: “I feel nostalgic at the moment,” “Right now, I am having nostalgic feelings,” “Right now, I am feeling quite nostalgic” (1 = *strongly disagree*, 4 = *strongly agree*; alpha = 0.95).

The mortality salience manipulation followed. We adapted the Word Relationship Task introduced by Yanagisawa and colleagues^23^ in their fMRI experiment. We created our own stimuli. In particular, we grouped 16 (Chinese) words into two categories of eight words each: death-related and neutral. We present relevant details in Supplementary Material. We informed participants that the task concerned perceptions of word relationships. A pair of words was presented in rapid succession (a total of 88 trials), and participants indicated whether the two paired words belonged to the same semantic category or to two different semantic categories by pressing “F” or “J”, respectively. Participants viewed: (1) 28 pairs of death-related words (*Same Category, death pairs*) selected from all combinations of the pool of eight death-related words (${\text{C}}_{{8}}^{{2}}$ = 28 combinations); (2) 28 pairs of neutral words (*Same Category, neutral pairs*) selected from all combinations of the pool of eight neutral words; and (3) 32 pairs of one death-related word and one neutral word, selected at random from the same word pools while avoiding identical pairs (*Different Category, control pairs*). In accordance with prior practice^23^, we did not analyze the control pairs (i.e., Different Category).

**Figure 3:**
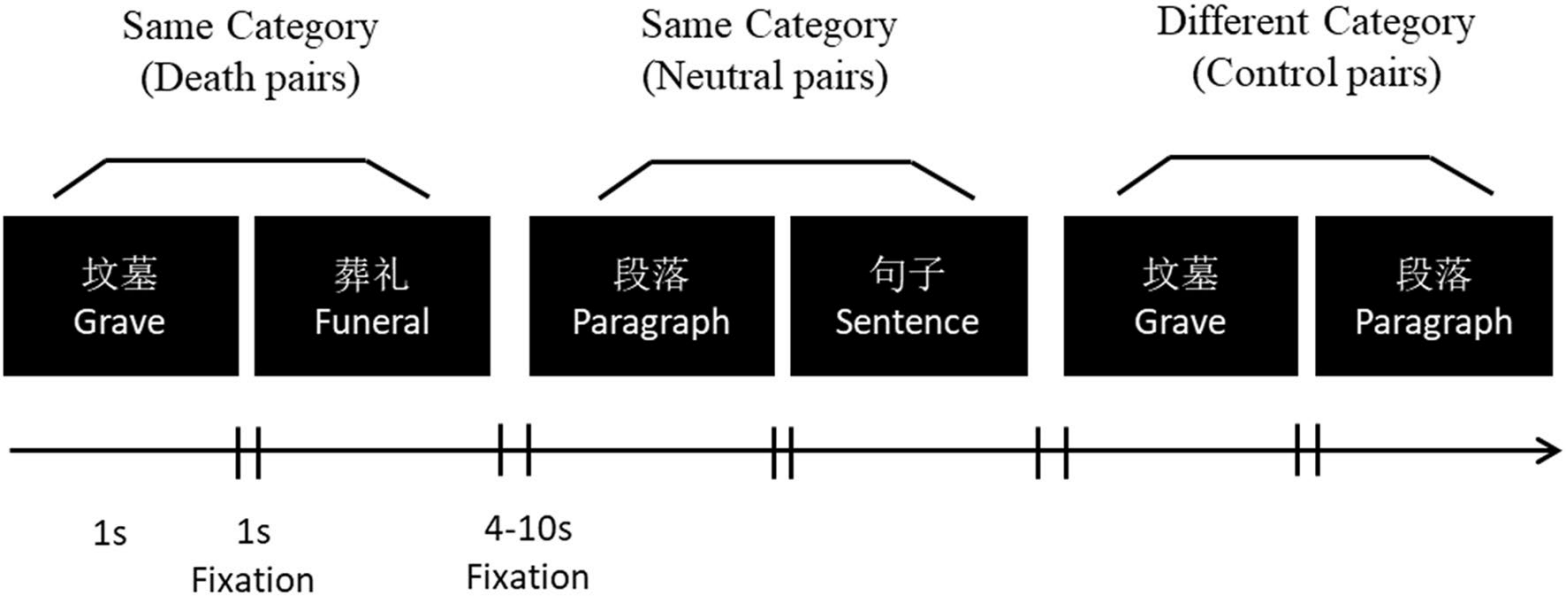
Word relationship task: participants judged as fast and accurately as they could whether two words presented in rapid succession belonged to the same category.

Each word was presented for 1 s. The interval between word pairs was 1 s, during which a fixation cross appeared on the screen. The intervals between word pairs ranged from 4 to 10 s, a practice used to maximize the efficiency of event-related designs. We instructed participants to respond as rapidly and accurately as possible. We depict the Word Relationship Task in Fig. 3. All stimuli were presented using Inquisit 4.0.

### Data acquisition

We conducted whole-brain imaging via a 3.0-Tesla MRI scanner (Discovery MR750, GE, USA). We acquired functional images using a gradient echo, echo-planar imaging (EPI) with the following parameters: repetition time (TR) = 2,000 ms, echo time (TE) = 30 ms, flip angle = 90°, acquisition matrix = 64 × 64, field of view = 220 mm, number of axial slices = 36, slice thickness = 3.5 mm, slice gap = 0.5 mm. We acquired a high-resolution (spatial resolution 1 × 1 × 1 mm) structural image using a T1-weighted, SPGR pulse sequence with the following parameters: flip angle = 8°, field of view = 256 mm, slice thickness = 1 mm. We placed firm padding around the head of each participant to restrict head motion. The visual stimuli were projected onto a screen and viewed through a mirror that was attached to a standard head coil.

### Data processing and statistical analyses

We preprocessed and analyzed functional data with the AFNI software package^44^. A standardized preprocessing pipeline for within-subjects analysis involved registration to the anatomy^45^, co-registration^46^, projection into standard stereotaxic space^47^, and smoothing with an 8 mm FWHM Gaussian kernel. We analyzed the ensuing preprocessed fMRI time series on a participant-by-participant basis using an event-related model in the context of voxel-wise multiple linear regression, with regressors for death-related and neutral words convolved with a canonical hemodynamic response function. Additionally, we included six motion parameters and the average signals extracted from white matter and cerebrospinal fluid as covariates of no interest. For each participant, we temporally indexed the activity associated with each experimental condition to the stimulus onset.

We presented participants with the modified version of the Word Relationship Task. We omitted from the analysis events for which participants made an incorrect response or gave no response. We calculated the parameter estimates (betas) for each condition for all brain voxels, and computed the relevant contrast (i.e., death-related vs. neutral) of the parameter estimates.

### Region of interest analysis

We conducted a hypothesis-driven region of interest (ROI) analysis on amygdala. We defined the left and right amygdala ROI based on the anatomical amygdala mask^47^ in ANFI (Fig. 1). We extracted the average percentage signal changes of all voxels within each of the left and right amygdala ROIs for death-related versus neutral pairs, and compared these using independent *t*-tests for the nostalgia versus control conditions (with Bonferroni correction for multiple comparisons).

### Whole brain analysis

We also conducted an exploratory whole brain analysis to examine how nostalgia would modulate brain responses to death-related (vs. neutral) stimuli. We incorporated the contrast between images of death-related versus neutral pairs into second-level group comparisons using a random effects model. For the whole-brain analyses, we set the threshold of significance to *p* < 0.05 (FDR correction for multiple comparisons). Given that the investigation of the neural basis of nostalgia is still in its infancy, we also ran whole brain analyses with a less strict threshold (*p* < 0.005, uncorrected for multiple comparisons)^48^. We reported the peak voxels of clusters that exhibited reliable effects in the MNI coordinates. We conducted anatomical identification by superimposing the maxima of the activation foci onto the MNI template, with the aid of the Anatomical Automatic Labeling Atlas^49^.

## Supplementary Information

Supplementary Information.

## Data Availability

The datasets generated during and/or analyzed during the current study are available from the corresponding author on request.

## References

[1] Leunissen JM, Wildschut T, Sedikides C, Routledge C (2020). The hedonic character of nostalgia: An integrative data analysis. Emot. Rev..

[2] Sedikides C, Wildschut T (2019). The sociality of personal and collective nostalgia. Eur. Rev. Soc. Psychol..

[3] Sedikides C (2015). To nostalgize: Mixing memory with affect and desire. Adv. Exp. Soc. Psychol..

[4] Hepper EG, Ritchie TD, Sedikides C, Wildschut T (2012). Odyssey’s end: Lay conceptions of nostalgia reflect its original Homeric meaning. Emotion.

[5] Holak SL, Havlena WJ (1998). Feelings, fantasies, and memories: An examination of the emotional components of nostalgia. J. Bus. Res..

[6] Wildschut T, Sedikides C, Arndt J, Routledge C (2006). Nostalgia: Content, triggers, functions. J. Pers. Soc. Psychol..

[7] Hepper EG, Wildschut T, Sedikides C, Robertson S, Routledge CD (2020). The time capsule: Nostalgia shields wellbeing from limited time horizons. Emotion.

[8] Madoglou A, Gkinopoulos T, Xanthopoulos P, Kalamaras D (2017). Representations of autobiographical nostalgic memories: Generational effect, gender, nostalgia proneness and communication of nostalgic experiences. J. Integr. Soc. Sci..

[9] Zhou X, Sedikides C, Wildschut C, Gao D-G (2008). Counteracting loneliness: On the restorative function of nostalgia. Psychol. Sci..

[10] Hepper EG (2014). Pancultural nostalgia: Prototypical conceptions across cultures. Emotion.

[11] Luo YLL, Liu Y, Cai H, Wildschut T, Sedikides C (2016). Nostalgia and self-enhancement: Phenotypic and genetic approaches. Soc. Psychol. Pers. Sci..

[12] Wildschut T, Sedikides C, Alowidy D (2019). *Hanin*: Nostalgia among Syrian refugees. Eur. J. Soc. Psychol..

[13] Sedikides C, Wildschut T (2018). Finding meaning in nostalgia. Rev. Gen. Psychol..

[14] Öhman A (2005). The role of the amygdala in human fear: automatic detection of threat. Psychoneuroendocrinology.

[15] Öhman, A., & Wiens, S. On the automaticity of autonomic responses in emotion: An evolutionary perspective in Series in affective science. In *Handbook of Affective Sciences* (eds. R. J. Davidson, K. R. Scherer, & H. H. Goldsmith) 256–275 (Oxford University Press, 2003).

[16] Mobbs D, Hagan CC, Dalgleish T, Silston B, Prévost C (2015). The ecology of human fear: Survival optimization and the nervous system. Front. Neurosci..

[17] Woody EZ, Szechtman H (2011). Adaptation to potential threat: The evolution, neurobiology, and psychopathology of the security motivation system. Neurosci. Biobehav. Rev..

[18] Carver CS, White TL (1994). Behavioral inhibition, behavioral activation and affective responses to impending reward and punishment: The BIS/BAS scales. J. Pers. Soc. Psychol..

[19] Stephan E (2014). The mnemonic mover: Nostalgia regulates avoidance and approach motivation. Emotion.

[20] FioRito TA, Routledge C (2020). Is nostalgia a past or future-oriented experience? Affective, behavioral, social cognitive, and neuroscientific evidence. Front. Psychol..

[21] Sedikides C, Wildschut T (2016). Past forward: Nostalgia as a motivational force. Trends Cogn. Sci..

[22] Sedikides C, Wildschut T (2020). The motivational potency of nostalgia: The future is called yesterday. Adv. Motiv. Sci..

[23] Yanagisawa K, Abe N, Kashima ES, Nomura M (2016). Self-esteem modulates amygdala-ventrolateral prefrontal cortex connectivity in response to mortality threats. J. Exp. Psychol. Gen..

[24] Cisler JM, Koster EH (2010). Mechanisms of attentional biases towards threat in anxiety disorders: An integrative review. Clin. Psychol. Rev..

[25] Phan KL, Wager T, Taylor SF, Liberzon I (2002). Functional neuroanatomy of emotion: A meta-analysis of emotion activation studies in PET and fMRI. Neuroimage.

[26] Bishop SJ (2008). Neural mechanisms underlying selective attention to threat. Ann NY Acad Sci..

[27] Liddell BJ (2005). A direct brainstem–amygdala–cortical ‘alarm’ system for subliminal signals of fear. Neuroimage.

[28] Feinstein JS, Adolphs R, Damasio A, Tranel D (2011). The human amygdala and the induction and experience of fear. Curr. Biol..

[29] Quirin M (2012). Existential neuroscience: A functional magnetic resonance imaging investigation of neural responses to reminders of one’s mortality. Soc. Cogn. Affect. Neur..

[30] Gamer M, Zurowski B, Büchel C (2010). Different amygdala subregions mediate valence related and attentional effects of oxytocin in humans. Proc. Natl. Acad. Sci. USA.

[31] Kirsch P (2005). Oxytocin modulates neural circuitry for social cognition and fear in humans. J. Neurosci..

[32] Klackl J, Jonas E, Kronbichler M (2013). Existential neuroscience: Neurophysiological correlates of proximal defenses against death-related thoughts. Soc. Cogn. Affect. Neur..

[33] Kessels LTE, Ruiter RAC, Jansma BM (2010). Increased attention but more efficient disengagement: Neuroscientific evidence for defensive processing of threatening health information. Health Psychol..

[34] Legault L, Al-Khindi T, Inzlicht M (2012). Preserving integrity in the face of performance threat: Self-affirmation enhances neurophysiological responsiveness to errors. Psychol. Sci..

[35] Liu X, Shi Z, Ma Y, Qin J, Han S (2013). Dynamic neural processing of linguistic cues related to death. PLoS ONE.

[36] Klackl J, Jonas E, Kronbichler M (2014). Existential neuroscience: Self-esteem moderates neuronal responses to mortality-related stimuli. Soc. Cogn. Affect. Neur..

[37] Sedikides, C., Skowronski, J. J., & Dunbar, R. I. M. When and why did the human self evolve? In *Evolution and Social Psychology: Frontiers in social Psychology* (eds. M. Schaller, J. A. Simpson, & D. T. Kenrick) 55–80 (Psychology Press, 2006).

[38] McGaugh JL (2004). The amygdala modulates the consolidation of memories of emotionally arousing experiences. Annu. Rev. Neurosci..

[39] Roozendaal B, McEwen BS, Chattarji S (2009). Stress, memory and the amygdala. Nat. Rev. Neurosci..

[40] Routledge C, Arndt J, Sedikides C, Wildschut T (2008). A blast from the past: The terror management function of nostalgia. J. Exp. Soc. Psychol..

[41] Faul F, Erdfelder E, Lang A-G, Buchner A (2007). G*Power 3: A flexible statistical power analysis program for the social, behavioral, and biomedical sciences. Behav. Res. Methods..

[42] Rosenberg, M. *The Measurement of Self-Esteem* (Princeton University Press 1965).

[43] Oba K, Noriuchi M, Atomi T, Moriguchi Y, Kikuchi Y (2016). Memory and reward systems coproduce ‘nostalgic’ experiences in the brain. Soc. Cogn. Affect. Neur..

[44] Cox RW (1996). AFNI: Software for analysis and visualization of functional magnetic resonance neuroimages. Comput. Biomed. Res..

[45] Saad ZS (2009). A new method for improving functional-to-structural MRI alignment using local Pearson correlation. Neuroimage.

[46] Cox RW, Jesmanowicz A (1999). Real-time 3D image registration of functional MRI. Magnet. Reson. Med..

[47] Talairach, J. Co-planar stereotaxic atlas of the human brain-3-dimensional proportional system. *An approach to cerebral imaging*. (1988).

[48] Lieberman MD, Cunningham WA (2009). Type I and Type II error concerns in fMRI research: Re-balancing the scale. Soc. Cogn. Affect. Neur..

[49] Tzourio-Mazoyer N (2002). Automated anatomical labeling of activations in SPM using a macroscopic anatomical parcellation of the MNI MRI single-subject brain. Neuroimage.

