# Supplementary material for: Nostalgia enhances detection of death threat: neural and behavioral evidence
Source: Sci Rep. 2021 Jun 16;11:12662. doi: 10.1038/s41598-021-91322-z (PMC8209061; doi:10.1038/s41598-021-91322-z)
Supplement: Supplementary file 1 — Supplementary Information. [file 41598_2021_91322_MOESM1_ESM.docx]

**SUPPLEMENTARY MATERIALS**

**Nostalgia Enhances Detection of Death Threat: Neural and Behavioral Evidence**

Ziyan Yang, Constantine Sedikides, Keise Izuma, Tim Wildschut,

Emiko S. Kashima, Yu L. L. Luo, Jun Chen, and Huajian Cai

**Stimulus Materials for the Nostalgia Manipulation**

We selected 100 pictures that we regarded as nostalgic, and 100 pictures that we regarded as non-nostalgic. In our selection, we relied on research indicating that childhood memories are particularly nostalgic (Hepper et al., 2012, 2014; Wildschut et al., 2006). As such, the nostalgic pictures depicted scenes or objects from childhood, whereas the control pictures depicted equivalent scenes or objects from modern life. For example, the nostalgic pictures depicted an elementary school classroom, a toy, an object (chewing gum), or a cartoon that referred to participants’ childhood. The control pictures, by contrast, depicted a contemporary classroom, toy, chewing gum, or cartoon. We resized all pictures to 640×480 pixels, and gave them a short title.

We tested 31 undergraduate students (20 women, 11 men), matched in age to the formal experiment’s sample (*M_age_* = 22.26 years, *SD_age_* = 2.26, *Range_age_* = 18-26 years), from 12 Beijing-based universities (e.g., Beijing Forestry University, Beijing Normal University, University of Chinese Academy of Sciences). We remunerated participants with 15 RMB ($2.20). Participants rated the 200 pictures for the extent to which they elicited nostalgia (1 = *not at all*, 5 = *extremely nostalgic*).

Based on these ratings, we selected 26 nostalgic pictures (each with an average nostalgicity score of ≥ 4), 26 control pictures (each with an average nostalgicity score of ≤ 2), and 26 baseline pictures (each with an average nostalgicity score of ≤ 2). Participants rated the nostalgic pictures (*M* = 4.39, *SD* = 0.42) as higher on nostalgicity than either the control pictures (*M* = 1.60, *SD* = 0.43), *t*(30) = 26.00, *p* < .001, or the baseline pictures (*M* = 1.56, *SD* = 0.47), *t*(30) = 23.84, *p* < .001. Their ratings of the control and baseline pictures did not differ significantly, *t*(30) = 1.23, *p* = .23.

**Stimulus Materials for the Mortality Salience Induction**

The stimuli for the Word Relationship Task consisted of eight death-related words (e.g., “kill,” “grave”) and eight neutral words (e.g., “word,” “phrase”). We tested 23 undergraduate students (13 women, 10 men), matched in age to the formal experiment’s sample (*M_age_* = 22.57 years, *SD_age_* = 1.75 years, *Range_age_* = 20-26 years), from nine Beijing-based universities (e.g., Beijing Forestry University, Beijing Normal University of Chinese Academy of Sciences). We paid participants 10 RMB ($1.50). They rated each word on (a) death relevance (1 = *not related at all*, 7 = *very strongly related*), (b) valence (1 = *extremely negative*, 7 = *extremely positive*), and (c) arousal (1 = *not arousing*, 7 = *very arousing*).

Results indicated that participants rated the death-related words (*M* = 6.58, *SD* = 0.88) higher on death relevance than the neutral words (*M* = 1.41, *SD* = 0.63), *t*(22) = 18.19, *p* < .001. Participants rated the death-related words (*M* = 2.26, *SD* = 0.94) lower on valence than the neutral words (*M* = 4.15, *SD* = 0.54), *t*(22) = -8.07, *p* < .001. Finally, participants rated the death-related words (*M* = 4.99, *SD* = 1.46) higher on arousal than neutral words (*M* = 2.52, *SD* = 1.27), *t*(22) = 6.67, *p* < .001.
